# Voronoi Decomposition of Cardiovascular Dependency Structures in Different Ambient Conditions: An Entropy Study

**DOI:** 10.3390/e21111103

**Published:** 2019-11-11

**Authors:** Dragana Bajic, Tamara Skoric, Sanja Milutinovic-Smiljanic, Nina Japundzic-Zigon

**Affiliations:** 1 Faculty of Technical Sciences-DEET, University of Novi Sad, Trg Dositeja Obradovica 6, 21000 Novi Sad, Serbia; tamara.ceranic@gmail.com or; 2 Faculty of Dental Medicine, University of Belgrade, Dr. Subotica 1, 11000 Belgrade, Serbia; sanja.milutinovic@stomf.bg.ac.rs; 3 School of Medicine, University of Belgrade, Dr. Subotica 1, 11000 Belgrade, Serbia or

**Keywords:** copula density, dependency structures, Voronoi decomposition, multiscale entropy, ambient temperature, telemetry, systolic blood pressure, pulse interval, thermoregulation, vasopressin

## Abstract

This paper proposes a method that maps the coupling strength of an arbitrary number of signals *D*, *D* ≥ 2, into a single time series. It is motivated by the inability of multiscale entropy to jointly analyze more than two signals. The coupling strength is determined using the copula density defined over a [0 1]*^D^* copula domain. The copula domain is decomposed into the Voronoi regions, with volumes inversely proportional to the dependency level (coupling strength) of the observed joint signals. A stream of dependency levels, ordered in time, creates a new time series that shows the fluctuation of the signals’ coupling strength along the time axis. The composite multiscale entropy (*CMSE*) is then applied to three signals, systolic blood pressure (*SBP*), pulse interval (*PI*), and body temperature (*t*_B_), simultaneously recorded from rats exposed to different ambient temperatures (*t*_A_). The obtained results are consistent with the results from the classical studies, and the method itself offers more levels of freedom than the classical analysis.

## 1. Introduction

Approximate [1,2] and sample entropies [3], *ApEn* and *SampEn*, have been intensively implemented in a range of scientific fields to quantify the unpredictability of time series fluctuations. Contributions that apply *ApEn* and *SampEn* are measured by thousands [4], confirming their significance. The cross entropies—*XApEn* and *XSampEn*—are designed to measure a level of asynchrony of two parallel time series [3,5,6]. Descriptions of (cross) entropy concepts can be found in numerous articles, but a recent comprehensive review [7] provides an excellent tutorial with the guidelines aimed to help the research society to understand *ApEn* and *SampEn* and to apply them correctly [7]. 

Multiscale entropy (*MSE*) [8,9], based on *SampEn*, investigates the changes in complexity caused by a change of the time scale. Composite *MSE* (*CMSE*) performs an additional averaging, thus solving the problem of decreased reliability induced by temporal scaling [10,11]. A comprehensive study of fixed and variable thresholds at different scales also presents an excellent review of the *MSE* improvements [12]. 

The benefits offered by entropy are explored in cardiovascular data analysis. Entropy was implemented to determine the cardiac variability [13], the complexity changes in cardiovascular disease [14], a level of deterministic chaos of heart rate variability (*HRV*) [9], *HRV* complexity in diabetes patients [15], in heart failure [16], in stress, [17,18] or in different aging and gender groups [19,20], while multiscale cross-entropy was applied for health monitoring systems [11].

*SampEn-* or *ApEn-*based entropy estimates are designed for one signal, or at most for two signals (cross-entropy), but biomedical studies often require an analysis of three or more simultaneously recorded signals. 

We propose a method that maps levels of interaction of two or more time series into a single signal. Levels of interaction are assessed using the copula density [21]. The transformation from the probabilistic copula domain to the beat-to-beat time domain is performed by Voronoi decomposition. 

The method is applied to multivariate time series that comprises three simultaneously recorded signals: systolic blood pressure (*SBP*), pulse interval (*PI*), and body temperature (*t*_B_) recorded at different ambient temperatures (*t*_A_). It is well known that thermoregulation can affect cardiovascular homeostasis [22]. Analysis of heart rate (*HR*) and *SBP* in the spectral domain has shown that changes of ambient temperature modulate vasomotion in the skin blood vessels, reflected in the very-low-frequency range of *SBP* and reflex changes in *HR* spectra [23,24]. Thermoregulation is complex and involves autonomic, cardiovascular, respiratory as well as a metabolic adaptation [25,26,27,28]. The key corrector of blood pressure is the baroreceptor reflex (BRR). The disfunction of BRR is the hallmark of cardiovascular diseases with a bad clinical prognosis. Thus, evaluating its functioning is important not only for the diagnosis and prognosis of cardiovascular diseases but also for the evaluation of treatment. 

The aims of this study are:

1. To propose a method that enables an application of multiscale entropy to an arbitrary number of signals and to analyze the outcome;
2. To compare the results of the classical multiscale method and the proposed method when applicable, i.e., in a case of two-dimensional signals;
3. To test whether the proposed method recognizes the changes of dependency level (coupling strength, level of interaction) of joint multivariate signals in different biomedical experiments.

The paper is organized as follows: the experimental setting for signal acquisition is explained in Section 2.1, together with surrogate signals and artificially generated control signals. The signal pre-processing that ensures the reliability of the results is explained in Section 2.2. Section 2.3. shows the mathematical tools assembled to create the proposed method: it gives an introduction to the copula theory, it outlines copula advantages and applications, and it discusses the various procedures for density estimation to justify the preference of Voronoi decomposition. 

Section 3.1. shows the basic statistical analysis of the experimental *SBP*, *PI*, and *t*_B_ signals. For the sake of comparison, this section includes the outcomes of classical *(X)SampEn* and *CMSE* entropy analysis. Section 3.2. introduces the new signal, created by the proposed method, for a two-dimensional case (*SBP* and *PI* mapped into the new *D* = 2 signal) and a three-dimensional case (*SBP*, *PI*, and *t*_B_ mapped into the new *D* = 3 signal). In both cases, the *SBP-PI* offset (delay) is taken into account ranging from 0 to 5 beats [29]. The wide sense stationarity of the created signals is checked and the correction proposed. The signals’ statistical properties, in terms of skewness and kurtosis, are estimated and discussed. In Section 3.3., the entropy parameters are analyzed and the proper ones that ensure the reliable estimates are selected. Then, the results of experiments performed to justify the consistency with the classical methods (in cases when the comparison is possible) are presented. The results showing that the method recognizes the changes in the level of signal interaction in various experimental environments are presented as well. The results are discussed in Section 4 with respect to the aims of this paper. The same section gives the conclusion and the possibilities for further method applications. 

A brief description of well-known entropy concepts—*ApEn*, *SampEn, MSE*, and *CMSE*—is included in the Appendix A.

## 2. Materials and Methods 

### 2.1. Experimental Setting and Signal Acquisition

All experimental procedures conformed to Directive 2010/63/EU National Animal Welfare Act 2009/6/RS and Rule Book 2010/RS. The protocol was approved by the University of Belgrade Ethics review board (license n°323-07-10519/2013-05/2).

**Figure 1:**
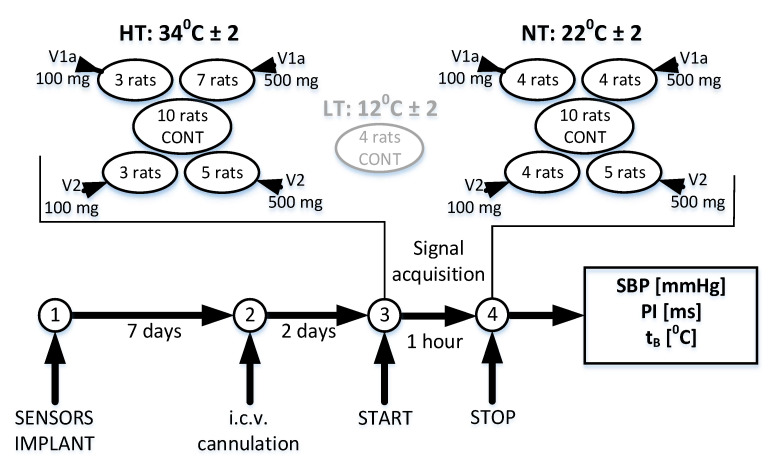
The experimental timeline and the signal subgroups. The high temperature (HT) experiment includes 28 animals exposed to 34 ± 2 °C ambient temperature; the neutral temperature (NT) experiment includes 27 animals exposed to 22 ± 2 °C ambient temperature; ten animals from each group were controls (CONT), the others got V1a and V2 antagonists, either 100 ng or 500 ng; the low temperature (LT) experiment contains four control animals exposed to 12 ± 2 °C ambient temperature; it is included as an illustration.

Adult male Wistar outbred rats, weighing 300–350 g, housed under control laboratory conditions (temperature—22 ± 2 °C; relative humidity: 60–70%; lighting: 12:12 h light-dark cycle) with food (0.2% NaCl) and tap water ad libitum were used in experimentation. Vasopressin selective antagonists of V1a or V2 receptors were injected via cannula chronically positioned in the lateral cerebral ventricle of the rat. The concomitant measurement of blood pressure waveforms (*BP*) and body temperature was performed using TL11M2-C50-PXT (DSI, St. Paul, MN, USA) equipment implanted into the abdominal aorta. The measurements were performed at the neutral ambient temperature (NT), 27 rats at *t*_A_ = 22 ± 2 °C, and the increased ambient temperature (HT), 28 rats at *t*_A_ = 34 ± 2 °C. The four rats recorded at the low temperature (LT), *t*_A_ = 12 ± 2 °C, were included as an illustrative example. There are five subgroups in NT and HT groups: control group, V1a-100 ng, V1a-500 ng, V2-100 ng, and V2-500 ng. The experimental timeline is shown in Figure 1. 

The experimental environment includes two types of control signals. The first controls are isodistributional surrogate data [30,31]. Surrogate data are derived from the experimental time series by randomizing the property that needs to be tested, keeping the other signal attributes intact. Thus, isodistributional surrogates randomly permute the signal to destroy the orderliness that is checked by entropy analysis. The signal distribution function remains unchanged. The second controls are artificially generated signals—a series of independent and identically distributed (i.i.d.) samples with Gaussian distribution and with exponential distribution. Gaussian signals possess a unique property in which linear independency implies statistical independency [32], and that it is an asymptotic distribution of the sum of i.i.d. samples (with some constraints) [32], an issue important for the multiscale entropy coarse-graining. Signals with exponential distribution are often implemented when there is a need to test the signals with large variance.

### 2.2. Signal Pre-Processing

Arterial blood pressure (*BP*) and body temperature signals were acquired using a sampling frequency of 1000 Hz. Systolic blood pressure (*SBP*) and pulse interval (*PI*) time series were derived from the *BP* waveforms as the local maxima and as the intervals between the successive maximal *BP* positive changes, respectively. The samples from the body temperature signals were taken simultaneously with *SBP* to create body temperature beat-to-beat time series *t*_B_. Artifacts were detected semi-automatically using the filter [33] adjusted to the signals recorded from the laboratory rats. A visual examination was then performed to find the residual errors. A very low signal component (trend) was removed by a high-pass filter designed for biomedical time series [34], thus ensuring *SBP*, *PI*, and *t*_B_ signal stationarity. All the signals were cut to the length of the shortest time series, *n* = 14,405 samples. The time series *X*_1_ = *SBP*, *X*_2_ = *PI* and *X*_3_ = *t*_B_ jointly create a single three-dimensional signal (*D* = 3). Its samples *X*_1*k*_, *X*_2*k*_, and *X*_3*k*_, *k* = 1,…, *N* create points in the three-dimensional signal space.

### 2.3. Copula Density, Voronoi Regions and Dependency Time Series

A copula is a mathematical concept that provides a multidimensional probability density function, where density reflects the level of signal interaction (dependency, coupling). It is introduced in 1959 [21] as a multivariate distribution function with marginals uniformly distributed on [0 1]*^D^*. If *X*_1_, …, *X_D_* are the source signals with joint distribution function *H* and univariate marginal distribution functions *F*_1_,…, *F_D_*, then copula *C* is defined as [21]:

(1) $$H\left(X_{1},\ldots ,\;X_{D}\right)=C\left(F_{1}\left(X_{1}\right),\ldots ,F_{D}\left(X_{D}\right)\right)$$

and vice versa:

(2) $$C\left(U_{1},\ldots U_{D}\right)=H\left(F_{1}^{-1}\left(U_{1}\right),\ldots ,F_{D}^{-1}\left(U_{D}\right)\right).$$

Sklar’s theorem [21] states that any *D*-dimensional joint distribution *H* with arbitrary univariate marginals could be decomposed into *D* independent uniform marginal distributions, bound together by a new joint distribution function *C*, called copula. 

The concept of the copula is based on the classical transformation of a random variable. Any continuous variable $X_{i}$ with a distribution function $F_{i\;}(X_{i}$) and density $f_{i\;}\left(X_{i}\right)\;=\frac{d\;F_{i\;}\left(X_{i}\right)\;}{dX_{i\;}},\;\;i=1,\ldots ,D\;$ can be transformed using a monotone function $U_{i\;}=\varphi_{i\;}\left(X_{i\;}\right)$. The result is a variable $U_{i}$ with a probability density function [32] $u_{i}(U_{i\;})=\frac{\;f_{i}(X_{i\;})}{\left|d\left(\varphi_{i\;}\left(X_{i\;}\right)\right)/dX_{i\;}\right|},\;\;X_{i\;}=\;\varphi_{i\;}{}^{-1}\left(U_{i\;}\right).$ The transformation function $\varphi_{i\;}\left(X_{i}\right)$ that creates a copula is the distribution function $F_{i}\left(X_{i\;}\right)\;$ of the signal $X_{i}$, i.e., $\varphi_{i\;}\left(X_{i\;}\right)=\;F_{i}\left(X_{i}\right)$. The new variable $U_{i}$ is then defined in [0, 1], as the following holds: $0\leq F_{i}\left(X_{i\;}\right)\leq 1$. It can be easily shown that the probability density function (PDF) of the new variable $U_{i\;}$ is uniform:

(3) $$u_{i}(U_{i\;})=\frac{\;f_{i}(X_{i\;})}{\left|d\left(F_{i}\left(X_{i\;}\right)\right)/dX_{i\;}\right|}=\frac{\;f_{i}(X_{i\;})}{\;f_{i}(X_{i\;})}=1.$$

The transformation of a random variable using its distribution function is known as probability integral transform, PI-transform, or PIT [35], and it is a core of the copula theory. It should be noted that the distribution function of a continuous variable, by definition, monotonically increases so the denominator in Equation (3) is positive, comprising just a single term.

The copula has been intensively used for the analysis and prediction of financial time series and the prediction of insurance risk [36,37,38,39], in hydrology and climate analysis [40,41,42] and communications [43]. Medical applications include aortic regurgitation study [44] and diagnostic classifiers for neuropsychiatric disorders [45]. A possibility to use a bivariate copula to analyze the cardiovascular dependency structures was introduced in [46] and pharmacologically validated by blocking the feedback paths using Scopolamine, Atenolol, Prazosin, and Hexamethonium. It was shown that Frank’s copula is the most appropriate to quantify the level of dependency of cardiovascular signals. 

**Figure 2:**
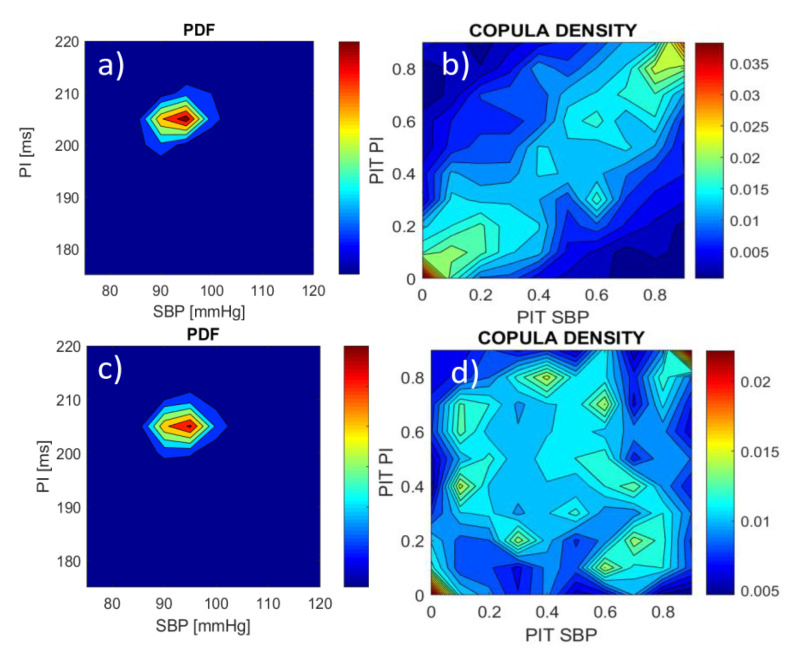
Bivariate probability density function (PDF) of *SBP* and *PI* signals and copula density of probability integral transformed (PIT) signals; (**a**,**b**) the offset between *PI* and *SBP* is equal to two beats; (**c**,**d**) the offset between *PI* and *SBP* is equal to ten beats; note that the PDFs in (**a**,**c**) are almost the same in spite of different *SBP-PI* offsets, while the copula density exhibits a strong positive dependency when offset is small (**b**), and a lack of dependency when offset is large (**d**).

Copula density $c\left(U\right)=\frac{\partial^{N}C\left(U_{1},\cdots ,\;U_{D}\right)}{\partial U_{1}\cdots \;\partial U_{D}}$ is used to visualize the intensity of signal coupling. The regions of increased copula density indicate the regions where the dependency of the signal samples increases. The difference between a classical bivariate probability density function (PDF) and the corresponding copula density of *SBP* and *PI* signals is that PDF shows the distribution of amplitude levels, while copula density shows the distribution of coupling strength between these amplitudes, regardless of the absolute amplitude values. An illustration of this difference is shown in Figure 2. *SBP* and *PI* signals and their probability integral transformed (PIT) counterparts are separated in time, first by two heartbeats (*SBP_k_* is coupled with *PI_k_*_+ 2_, *k* = 1, 2, …, *N−*2), and then by ten heartbeats (*SBP_k_* is coupled with *PI_k_*_+ 10_, *k* = 1, 2, …, *N−*10). The copula density in panel b exhibits a distinct linear positive coupling structure that follows the known physiological relationships [47]. The copula density in panel d shows almost uniform distribution as the time offset between *SBP* and *PI* signals is sufficiently large to attenuate their mutual dependency. Contrary to copula density, the joint probability density functions are almost the same in both cases (panels a and c). The temporal separation of *SBP* and *PI* signals does not alter the mutual relationship of signal amplitudes, but it significantly alters the intensity of signal coupling. 

The advantages of copula are numerous. Copula density visualizes the dependency structures of the observed signals, and it quantifies the signal coupling strength (“copula parameter”). It captures both linear and nonlinear relationships between the signals. It can quantify the intensity of signal coupling within the different regions of the copula domain, and, in particular, it can model the tail dependencies of the signals. 

Such a visualization, in a case of *SBP-PI* signals, cannot be achieved by other methods: the Oxford method, the oldest and the referent procedure for the evaluation of the baroreceptor reflex, uses increasing doses of short-acting vasoconstrictors (e.g., phenylephrine) and vasodilators (e.g., nitroprusside) to trigger heart deceleration of acceleration. The *SBP* and *PI* relationship is plotted as a fitted sigmoid curve. It is an invasive method, and it does not show spontaneous BRR. The most acknowledged among the non-invasive approaches is the sequence method, with the visualization that shows the scatterplot of the signal points that are elements of BRR sequences (i.e., the scatterplot contains a subset of all signal points). The method quantifies the spontaneous BRR operating range and set point [48], but the visualization is similar to the classical probability density function. 

The time offset (delay) between *SBP* and *PI* signals is important for the signal coupling, and it depends on species. It was shown [49] that the delay of 0, 1, and 2 beats is the most appropriate for humans, while the delays of 3, 4 and 5 beats are appropriate for rats [29] and mice [47]. In [50], it was shown that, in laboratory rats, the highest level of comonotonic behavior of pulse interval and systolic blood pressure is observed at time lags 0, 3, and 4 beats, while a strong counter-monotonic behavior occurs at time lags of 1 and 2 beats. 

Copula density is a probabilistic quantity. To convert it into a time series, to each point in the time domain, an appropriate density (dependency level) $DL_{k}$ should be assigned, thus creating a dependency signal $DL_{k},k=1,\ldots ,\;N$.

A trivial way to estimate a copula density is to create a *D*-dimensional histogram. The obtained $DL_{k}$ signal would be discrete, as the points within the same histogram bins would get the same value. An increased number of histogram bins would increase the number of discrete signal levels, but the estimation reliability would decrease. 

Density estimation based on Markov chains [51] creates a stochastic matrix of “transition probabilities”—scaled distances—between the points, with the steady-state probabilities proportional to the required distribution. The method is computationally inefficient in multidimensional space, except for the short time series. 

A *D*-dimensional sphere (or cube) around a particular signal point defines a local density according to the number of encircled neighbors. The procedure is efficient, but the neighboring spheres overlap inducing the bias, and the result depends on the sphere diameter (i.e., threshold) choice. 

The chosen approach expresses the sample density proportionally to the non-overlapping free space surrounding the sample. Such a concept has long been known as the Voronoi region. It can be traced back to the scholars from the 17th and 18th centuries, but it was re-discovered, analyzed, and its applications outlined at the beginning of the 20th century [52]. 

The concept is simple: Let A be the set of all points in a [0 1]*^D^* copula space. Let $U_{k}=[U_{1k},\ldots ,\;U_{Dk}],\;\;k=1,\;\ldots ,\;N$ be a *D*-dimensional point from a PI-transformed multivariate time series. Then, the Voronoi region $R_{k}^{D}$ around the point $U_{k}$ comprises all the points from A that are closer to the particular point $U_{k}$ then to any other point $U_{j},\;\;j=1,\;\ldots ,\;N,\;j\neq k.$ More formally,

(4) $$R_{k}^{D}=\{a\in A\;|\;d\left(a,U_{k}\right)\;\leq d\left(a,U_{j}\right),\;\;\forall \;j\;\neq k\}.$$

A classical Euclidean distance is typically chosen for the distance $d\left(a,\;U_{k}\right)$, but any other distance measure can be used as well, resulting in different Voronoi decompositions. 

**Figure 3:**
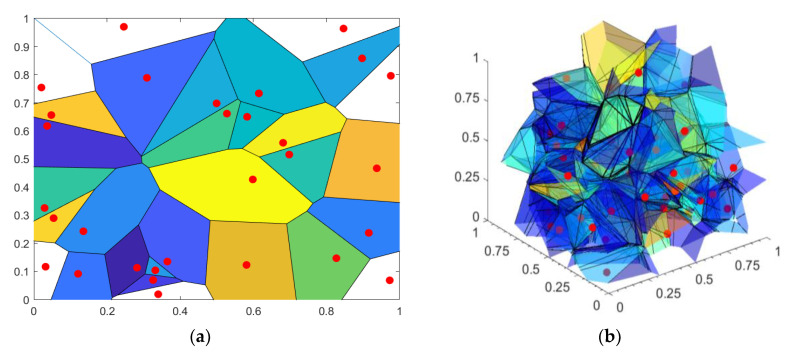
Voronoi region (polytope) and the corresponding signal points. (**a**) An example of Voronoi cells in a two-dimensional plane (*D* = 2); (**b**) An example of Voronoi polyhedrons in three dimensions (*D* = 3). The uncolored cells/polyhedrons both in (**a**) and (**b**) are cut to fit the [0 1]*^D^* space.

Figure 3 shows examples of the Voronoi regions in two and three dimensions. The line segments that separate particular Voronoi cells $R_{k}^{2}$ and $R_{j}^{2}$ in the left panel of Figure 3 are the sets of the points a∈A   that are equidistant to the points $U_{k}$ and $U_{j}$, i.e., $d\left(a,\;U_{k}\right)=d\left(a,U_{j}\right).$ The Voronoi vertex a∈A   in the same panel is the point equidistant to three (or more) time series points, e.g., $d\left(a,\;U_{k}\right)=d\left(a,\;U_{j}\right)=d\left(a,\;U_{l}\right)$. The right panel (*D* = 3) also shows Voronoi lines and vertices, but, in the [0 1]^3^ domain, this is more difficult to visualize. Uncolored Voronoi regions are either unbounded, or the boundaries are outside the [0 1]*^D^* space. These regions are cut to fit the [0 1]*^D^* space. 

A series of surface areas in two-dimensional Voronoi regions and a series of volumes in three-dimensional Voronoi regions are a good foundation to quantify the dependency level and to form the time series $DL_{k},k=1,\ldots ,\;N,\;\mathrm{as}$:

(a) The surface/volume of $R_{k}^{D}$ is inversely proportional to the dependency level of the point $U_{k}$. An increased density of dependency structures in [0 1]*^D^* space implies a decrease of available space between the points.
(b) The region $R_{k}^{D}$ is shaped like the best distance separation of the point $U_{k}$, so its surface/volume is unambiguously calculated and unique, without a necessity to include any thresholds.

The drawback of the method is that a change of distance measure changes the shape of regions. We have opted for Euclidian distance as a classical approach for distance measurement, widely used in a wide range of applications.

## 3. Results

### 3.1. Source Signal Analysis

The total number of *SBP-PI-t*_B_ signal triplets is equal to 59. The basic statistical parameters, shown as a control, are presented in Table 1. Results in Table 1 show no significant changes in statistical parameters of *SBP*, *PI*, and *t*_B_ signals. An earlier study [26] revealed that V1a antagonists increase body temperature. The differences might be the outcome of different measurement procedures: in this study, the temperature is measured using a telemetric probe in the abdominal aorta, while, in [26], the temperature was measured rectally. 

**Figure 4:**
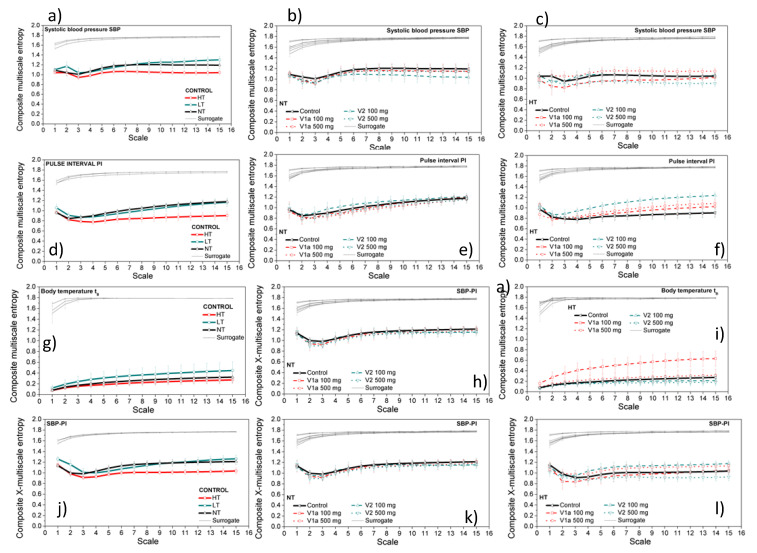
Composite multiscale entropy estimated from the source signals. From top to bottom, entropy was applied to *SBP, PI*, t_B_ signals, and *SBP-PI* signal pairs. Left panels: entropy of the control signals at different ambient temperatures; middle panels: effects of antagonists at neutral temperature; right panels: effects of antagonists at high temperature. Results are presented as a mean ± SE (standard error).

Figure 4 shows the results of the classical entropy analysis, performed for the sake of comparison. The left panels show the *CMSE* of the signals recorded from control animals at different ambient temperatures. The middle panels show the effect of drugs at the neutral temperature. The left panels show the effects of drugs at a high temperature. Each signal is accompanied by ten isodistributional surrogate signals, generated by a random temporal permutation of the signal samples [30,31]. 

The first three rows in Figure 4 show the classical composite multiscale entropy analysis of a single-dimensional time series, *SBP*, *PI*, and *t*_B_, respectively. The last row shows multiscale *SBP-PI* cross-entropy that can be compared to the multiscale entropy of the new signals. 

### 3.2. Properties of the Dependency Time Series

The created time series are new signals, so their statistical properties need to be checked before entropy analysis.

Mapping the signals into the dependency time series takes into account the delay (offset) between the *PI* and *SBP* signals. The time delay (offset) DEL=0,⋯, 5 [beats] applied to each pair of *SBP*-*PI* signals resulted in six two-dimensional (2D) time series $\left(SBP_{k},\;PI_{k+DEL}\right)$, and six three-dimensional (3D) time series $\left(SBP_{k},\;PI_{k+DEL},t_{Bk}\right),\;\;k=1,\ldots ,\;N-DEL.$ The total of 354 *SBP-PI* pairs and 354 *SBP-PI-t*_B_ triplets were converted into two-dimensional and three-dimensional Voronoi cell time series. An average percentage of Voronoi cells that had to be cut to fit the [0 1]*^D^* space was 2.68% for two-dimensional, and 16.26% for three-dimensional signals (cf. Figure 3). Additionally, 11 signal points (0.0002%) were too close to the vertices of the [0 1l^3^ cube to generate the three-dimensional polyhedrons, so they were managed manually. 

**Figure 5:**
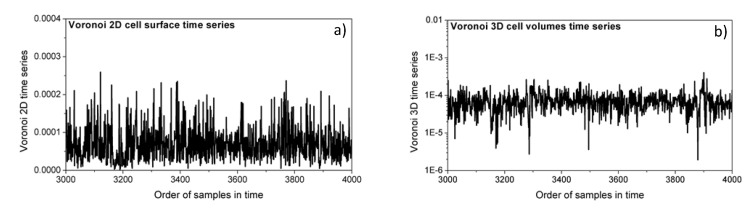
Samples of Voronoi cells time series. (**a**) two-dimensional signals (*SBP* and *PI* interaction, *D* = 2); (**b**) three-dimensional signals (*SBP*, *PI* and *t*_B_ interaction, *D* = 3).

Examples of Voronoi cell time series are shown in Figure 5.

A wide sense stationarity (WSS) test [53] is then applied, as the stationarity is an obligatory prerequisite for entropy estimation [7,38]. The test checks the stationarity of the first and the second statistical moments. The three-dimensional Voronoi cells time series failed the second-moment test.

**Figure 6:**
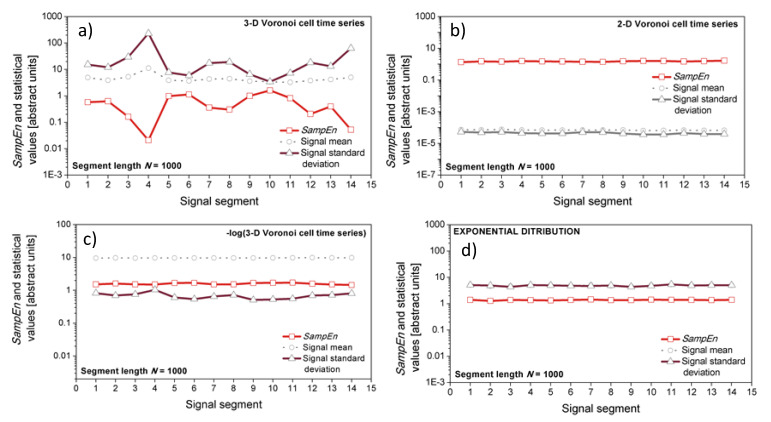
*SampEn*, mean and variance of a time series. Note the high variability of entropy estimated in the different segments of three-dimensional Voronoi cells time series (**a**) (*SBP*, *PI* and *t*_B_ interaction, *D* = 3), smoothed by logarithm; (**c**) two-dimensional Voronoi cells time series (*SBP* and *PI* interaction, *D* = 2) are stationary (**b**) as well as the signal created from the interaction of three exponentially distributed random signals, *D* = 3 (**d**).

Figure 6 shows the negative effects of non-stationarity: a three-dimensional non-stationary Voronoi cell time series is cut into 14 successive segments, each one comprising *n* = 1000 signal points. Then, mean, variance, and entropy were estimated from each segment and plotted in Figure 6a. Figure 6b shows the same parameters but estimated from the two-dimensional stationary Voronoi cells time series. 

The difference between two- and three- dimensional signals is a consequence of coverage. The number of signal points in two-dimensional space is sufficient to ensure good coverage. The same points are sparsely and unevenly scattered in three-dimensional space, so the estimation is unreliable, resulting in different values obtained from the different sections of the same signal. 

Panel d in Figure 6 shows the results from artificially generated time series with exponentially distributed samples. It is a usual control example of a signal with large, but time-invariant, variance. It passed the stationarity test and the parameters estimated from it are constant in each segment. 

Taking a logarithm is a procedure that ensures the stationarity of the second moment. A negative logarithm corresponds to the inverse of the Voronoi cell volume, and it is proportional to the local sample density. It is always positive as the inverse of any Voronoi cell volume in [0 1]*^D^* domain is greater than 1. Panel c of Figure 6 is a visual confirmation of a successful test outcome. 

The dependency level time series, *DL*, is finally defined as the negative logarithm of the Voronoi cell time series. The number of signal points (*N* = 14,400) ensures the signal stationarity at least in the wide sense for two-dimensional (*D* = 2) and three-dimensional (*D* = 3) signals. 

**Figure 7:**
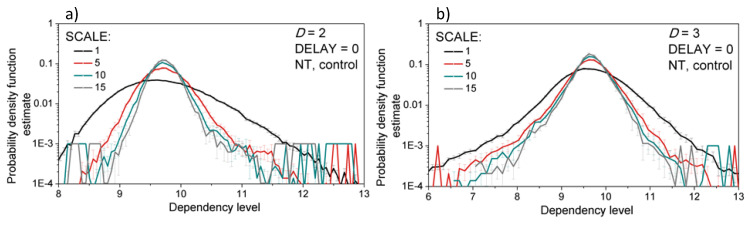
Empirical probability density function of the created signals, averaged over 10 control rats at neutral temperature (NT). (**a**) two-dimensional signals (*SBP* and *PI* interaction, *D* = 2); (**b**) three-dimensional signals (*SBP*, *PI* and *t*_B_ interaction, *D* = 3). The *SBP-PI* offset (DELAY) is equal to 0 beats. Results are presented as a mean ± SE (standard error).

**Figure 8:**
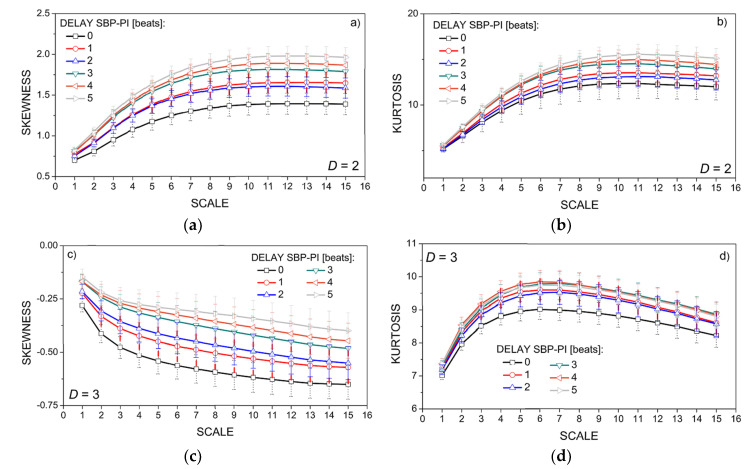
Skewness (panels (**a**,**c**)) and kurtosis (panels (**b**,**d**)) for different SBP-PI offset (DELAY), averaged over all 59 created signals; panels (**a**,**b**) two-dimensional signals (*SBP* and *PI* interaction, *D* = 2); panels (**c**,**d**) three-dimensional signals (*SBP*, *PI*, and *t*_B_ interaction, *D* = 3); results are presented as a mean ± SE.

The statistical properties of the new signals—probability density function, skewness, and kurtosis—are shown in Figure 7 and Figure 8.

Empirical probability density functions for different scaling levels are plotted in Figure 7. Signals are normalized and centered so the changes of mean and variance due to the convolution are not visible. There is no significant difference in the estimated probability density functions for the scaling levels greater than five.

Skewness is a third statistical moment that shows a level of signal asymmetry around the mean. The skewness of the two-dimensional dependency signal (*SBP* and *PI* interaction) is presented in Figure 8a). It is positive, with a right tail exhibited, indicating the existence of signals with a strong dependency level between systolic blood pressure and a pulse interval. The positive skewness increases with the increasing offset (delay) *DEL* between *SBP* in *PI*. It is in accordance with [29] that located the dominant *SBP-PI* relationships at offset of 3, 4, and 5 beats. 

The skewness of the three-dimensional dependency signal (*SBP*, *PI*, and *t*_B_ interaction) is presented in Figure 8b). It is close to zero—slightly negative—so the level of the dependency between the three signals is almost symmetric. It may indicate that the inclusion of body temperature into the new signal attenuates the *SBP-PI* signal coupling. The increase of the *SBP-PI* offset (delay) *DEL* results in the increased skewness shifted closer to zero, towards the positive values, again in accordance with [29]. 

Kurtosis measures the intensity of probability density function “tails”. It is shown in Figure 8b for two-dimensional signals (*SBP* and *PI* interaction) and in Figure 8d for three-dimensional signals (*SBP*, *PI*, and *t*_B_ interaction). The tails of dependency signals are heavy if compared to Gaussian distribution, indicating an increased number of signals with very high and very low dependency levels. It is expected, due to the high variance of three-dimensional dependency signals. The intensity of tails increases with the increased *SBP-PI* offset *DEL*, and it also increases with an increase of scale. This is also expected, as scaling convolves the probability density functions of the components that are coarse-grained. The convolution emphasizes the tail parts of the distribution in spite of the normalization, as the convolved samples are not Gaussian. 

### 3.3. Entropy Analysis of the Dependency Time Series

Entropy is a parametric method, with parameters determined to ensure its reliable estimation. The statistical analysis from Section 3.2, however, is insufficient to provide the guidelines for entropy parameter selection for the new dependency time series. 

**Figure 9:**
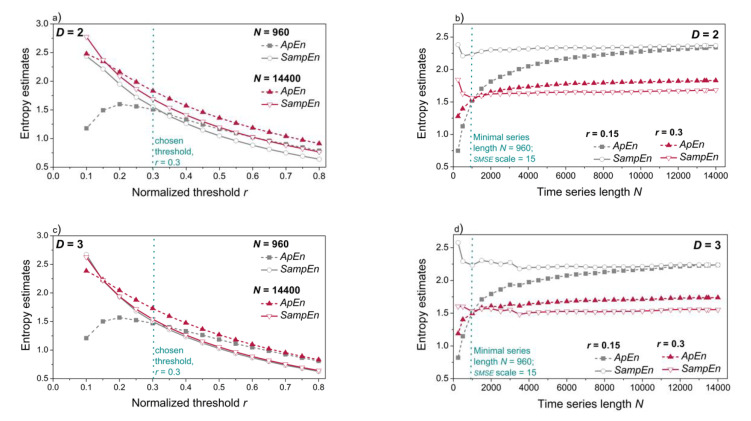
Threshold profile (panels (**a**,**c**)) and length profile (panels (**b**,**d**)) of a single subject; vertical dashed lines mark the chosen threshold *r* = 0.3 in panels (**a**,**c**) and minimal series length *n* = 960 (the highest scale) in panels (**b**,**d**); upper panels: two-dimensional signals (*SBP* and *PI* interaction, *D* = 2); lower panels: three-dimensional signals (*SBP*, *PI* and *t*_B_ interaction, *D* = 3.

It has already been pointed out [18,54,55,56,57,58,59] that the threshold (filter) *r* (cf. Equation (A3)) is one of the major causes of inconsistency in entropy estimation and that its choice is related to the series length *N*. Thus, the threshold and length profiles of the dependency time series are plotted in Figure 9. Although the multiscale entropy is defined on the *SampEn* basis, the figure also includes *ApEn* as the worst-case example. 

In a multiscale entropy approach, the time series length step-wise decreases with the increased scaling level. Maximal series length of our signals is equal to *n* = 14,400. If the scaling level is equal to 15, then the minimal series length would be equal to *n* = 960. Panels a and c of Figure 9 show the threshold profile of *ApEn* and *SampEn* for the maximal and for the minimal lengths. Stable results are achieved for threshold *r* = 0.3 [18]. 

The length profile is plotted in panels b and d for the typical threshold value *r* = 0.15 and the chosen threshold value *r* = 0.3. It can be seen that the results are not consistent for lengths below *n* = 900, so the choice of 15 scaling levels is justified. 

**Figure 10:**
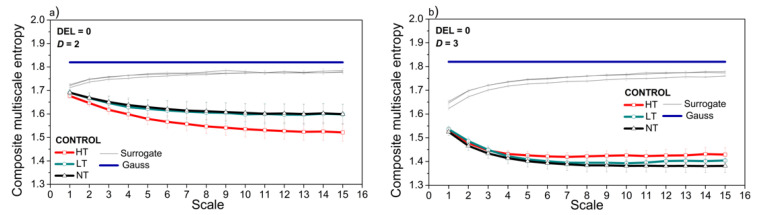
Comparison of CSME estimates for signals recorded from control animals at high ambient temperature (HT), low temperature (LT) and neutral temperature (NT). (**a**) two-dimensional signals (*SBP* and *PI* interaction, *D* = 2); (**b**) three-dimensional signals (*SBP*, *PI* and *t*_B_ interaction, *D* = 3). Delay (offset) of *SBP-PI* signals was set to DEL = 0 beats. Signals are accompanied by the control surrogate study and by the artificial two- and three- dimensional Gaussian signals. Results are presented as a mean ± SE.

**Figure 11:**
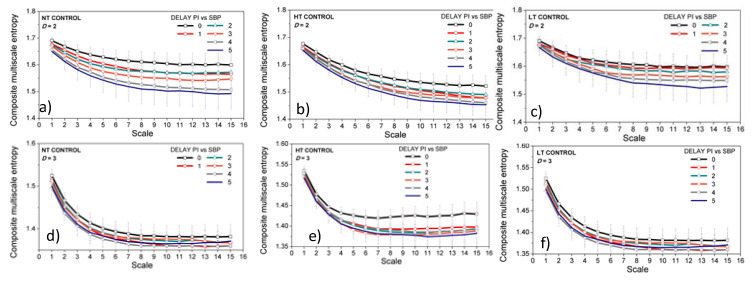
Composite multiscale entropy *CMSE* with *SBP-PI* offset (DELAY) as a parameter, estimated from control animals exposed to neutral temperature (NT, panels (**a**,**d**)), high temperature (HT, panels (**b**,**e**)), and low temperature (LT, panels (**c**) and (**f**)). Upper panels (**a**–**c**) two-dimensional signals (*SBP* and *PI* interaction, *D* = 2); lower panels (**d**–**f**) three-dimensional signals (*SBP*, *PI* and *t*_B_ interaction, *D* = 3). Results are presented as a mean ± SE. Statistically significant difference (*p* < 0.05) between the lowest and highest offsets, DEL = 0 and 5, are observed for the scale greater than 5 in panels (**a**–**c**,**e**).

**Figure 12:**
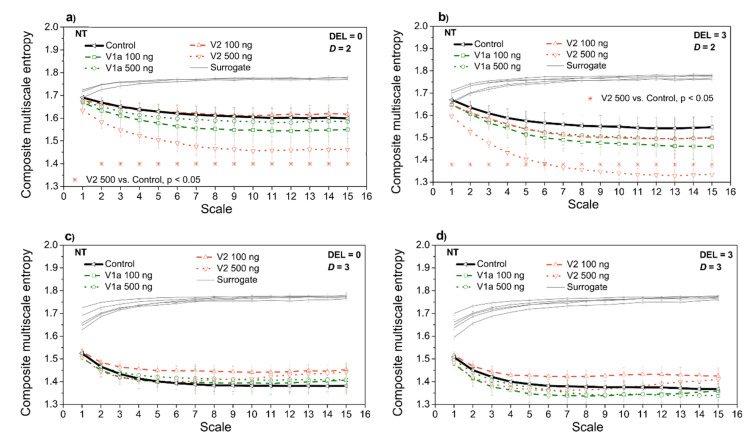
Composite multiscale entropy *CMSE* estimated from rats exposed to vasopressin antagonists at neutral temperature (NT). Panels (**a**,**c**) *SBP-PI* offset (delay) is set to DEL=0; panels (**b**,**d**) *SBP-PI* offset (delay) is set to DEL=3; Upper panels (**a**,**b**) two-dimensional signals (*SBP* and *PI* interaction, *D* = 2); lower panels (**c**,**d**) three-dimensional signals (*SBP*, *PI* and *t*_B_ interaction, *D* = 3). Results are presented as a mean ± SE.

**Figure 13:**
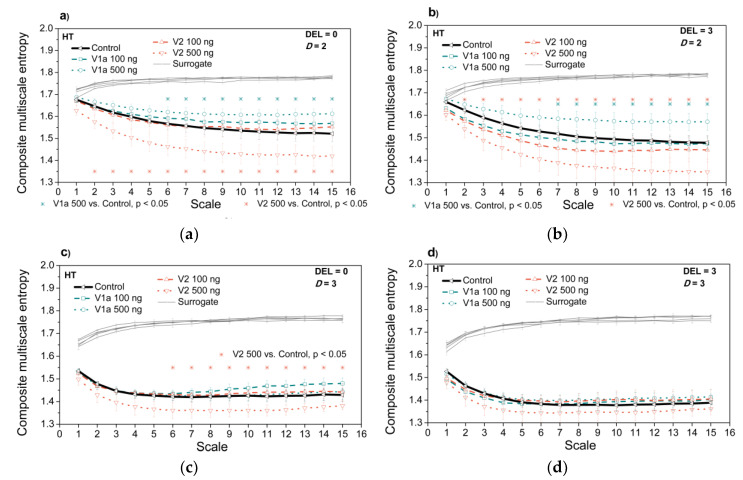
Composite multiscale entropy *CMSE* estimated from rats exposed to vasopressin antagonists at high temperature (HT). Panels (**a**,**c**) *SBP-PI* offset (delay) is set to DEL=0; panels (**b**,**d**) *SBP-PI* offset (delay) is set to DEL=3; Upper panels (**a**,**b**) two-dimensional signals (*SBP* and *PI* interaction, *D* = 2); lower panels (**c**,**d**) three-dimensional signals (*SBP*, *PI* and *t*_B_ interaction, *D* = 3). Results are presented as a mean ± SE.

**Figure 14:**
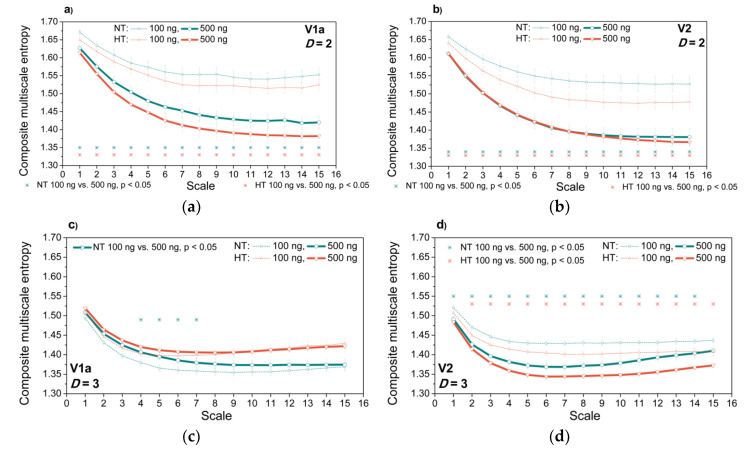
Effects of V1a and V2 antagonist dosage. Panels (**a**,**b**) two-dimensional signals (*SBP* and *PI* interaction, *D* = 2); panels (**c**) and (**d**) three-dimensional signals (*SBP*, *PI* and *t*_B_ interaction, *D* = 3). Panels (**a**,**c**) V1a antagonist; panels (**b**,**d**) V2 antagonist. Results are presented as a

Figure 10, Figure 11, Figure 12, Figure 13 and Figure 14 show the main result of the composite multiscale entropy study of dependency level signals, with the scaling level set to 15, and the threshold level set to *r* = 0.3. These results are discussed in Section 4. 

Figure 10a presents *CSME* estimated from the dependency level signals of control rats at different ambient temperatures, for two-dimensional signals (*SBP* and *PI* interaction, *D* = 2). Each signal is accompanied by 10 isodistributional surrogates (the estimated entropy is averaged). These results can be compared to the results of the classical entropy analysis shown in Figure 6j. The figure also presents *CSME* estimated from the artificially generated two-dimensional signals with Gaussian distribution. Figure 10b shows the same entropies but estimated from the three-dimensional signals.

The aim of Figure 11 is to show whether the *CMSE* estimated from the signals of control rats at different ambient temperatures can distinguish different temporal offsets (DEL) between the *SBP* and *PI*. Such an analysis cannot be performed by classical entropy study. 

Differences between experimental groups were analyzed by a Mann–Whitney U-test. Statistical significance was considered at *p* < 0.05.

Figure 12 presents the entropy estimates after the administration of vasopressin antagonists at neutral ambient temperature, for two different *SBP-PI* temporal offsets (delays), DEL = 0, and DEL = 3. Figure 13 presents the same entropy estimates but at the high ambient temperature. Both Figure 12 and Figure 13 are accompanied by isodistributional surrogate data controls. The purpose of these two figures is to investigate whether CMSE can distinguish the V1a and V2 antagonist administration if compared to the control case (without the drugs). The two-dimensional cases can be compared to the classical entropy study shown in Figure 4. 

The purpose of Figure 14 is to check whether the proposed entropy can distinguish the signals after administering the different doses of V1a and V2 antagonists. The same experiments are repeated separating the signals according to the *SBP-PI* offset (delay), and these results are presented as Supplementary data. 

## 4. Discussion

The first aim of our study is to create a single time series that reflects the level of interaction between the arbitrary number of simultaneously recorded signals. It is accomplished by mathematical tools: copula density captures the level of signal interaction, and the Voronoi decomposition maps the interaction levels into the temporal signal. 

Figure 10a shows that the *CMS* entropy of two-dimensional *SBP-PI* dependency signal decreases at high ambient temperature. The classical *CMSE* analysis provided the same result (Figure 4, panel j). The high ambient temperature in this experiment exceeds the boundary set to 29.5 °C [60], inducing heat dissipation in rats such as vasodilatation, evaporation, sweating, panting and affecting the blood vessel circulatory strain [60]. The existence of the dominant component reduces the influence of the other mechanism, the signals and their mutual interactions become less complex, and the entropy decreases. On the other hand, neutral and low ambient temperatures in our experiments are within the normal boundaries, and entropy estimates overlap, both in the proposed and classical entropy estimates—Figure 10a and Figure 4j—respectively. 

An administration of V2-500ng significantly decreases the multiscale entropy of a two-dimensional *SBP-PI* dependency signal, both at neutral (Figure 12a,b) and at high ambient temperature (Figure 13a,b). Additionally, V1a-500 significantly increases the entropy of a two-dimensional *SBP-PI* dependency signal, signals at high temperatures (Figure 13a,b). These analyses were performed at the particular offsets (delays) between *SBP* and *PI* signals. The results correspond to the spectral analysis of the signals after V1a and V2 antagonist administration: it was shown [27] that V2-500 ng administration increases the low-frequency signal component of the *SBP* signal; the signal becomes smoother, the number of repetitive patterns increases, and the signal becomes more predictive so the entropy decreases. On the other hand, V1a-500 ng increases the high-frequency signal component [27], the signal, and its interactions become more turbulent and the entropy increases. 

While spectral analysis separates high and low signal components, a classical cross-entropy observes the signal as a whole. The template matching procedure (cf. Equation (A3)) averages all the temporal *SBP* and *PI* positions, so different offsets (delays) of *SBP-PI* signals cannot be distinguished. Panels k and l of Figure 4 show that classical *SBP-PI* cross-entropy does slightly decrease after V2-500 ng administration, but without the statistical significance, especially at the neutral temperature (Figure 4k). 

It is consistent with the second aim of our contribution: the proposed analysis corresponds to the classical two-dimensional entropy results and with the results of spectral analysis. In cases when the consistency is not statistically significant, the possible explanation is that the classical entropy observes the complete signal, while the proposed method includes signals separated according to the SBP-PI signal offset. 

When the body temperature time series is included to create three-dimensional *SBP-PI-t*_B_ dependency signals, the entropy decreases and differences caused by ambient temperature are attenuated (Figure 10b). It should be noted (Figure 4g,h,j) that signal *t_B_* is a low-entropy signal. However, the significant entropy decrease induced by V2-500 ng is preserved in three-dimensional signals, but only at the high temperatures and DEL = 0 (Figure 13c). 

The proposed method can make a distinction between the different *SBP-PI* offsets (delays), as shown in Figure 11. Regardless of the ambient temperature, the entropy of the two-dimensional dependency signals at DEL = 5 is significantly lower than at DEL = 0. It corresponds to [29], as the *SBP-PI* dependency in rats is the greatest for delays of 3, 4, and 5 beats. When three-dimensional signals are observed, the significant decrease of entropy for DEL = 5 occurs at high ambient temperatures only. 

The proposed entropy can distinguish the signals after administering the different doses of V1a and V2 antagonists. It is shown in Figure 14. The distinction is statistically significant for the two-dimensional *SBP-PI* dependency signal, regardless of antagonist and ambient temperature, and for three-dimensional signals after V2 administration. V1a administration induces a statistically significant difference in three-dimensional *SBP-PI-t*_B_ dependency signal at the neutral temperature, and only for a couple of scaling levels. 

Figure 14 presents the entropy estimates averaged over all the signals. The Supplementary data comprise Figures S1 and S2, where the dosages are separated according to the *SBP-PI* offset (delay).

The entropy of the artificial two- and three-dimensional Gaussian control signals is shown in Figure 10, but not repeated in the subsequent figures as it is always the same. The entropy of the surrogate data converges towards the Gaussian, but never reaches it: although the surrogate signals can be regarded as streams of i.i.d. random variables, their distribution remains equal to the distribution of the original dependency signals. 

## 5. Conclusions

The signal framework created in this contribution provides a possibility for an easy analysis of signal dependency structures mapped into a single time series. The estimated entropies of two-dimensional dependency signals correspond to the classical cross-entropy and spectral analysis. The method can recognize entropy changes at different temperature levels, at different *SBP-PI* offsets, at different administered V1a and V2 dosages. The recognition of these differences is not random: the surrogate data analysis destroys the temporal coupling of the observed dependency signals, yielding in all cases entropy estimates that are almost identical and that approach (but do not reach) the entropy of Gaussian time series.

This method can be applied to any multivariate signals. It is necessary to conduct a deep analysis to find the minimal signal length that provides reliable results for the arbitrary number of signals and to check the possibility of applying other analytical tools besides the entropy. A comparative study of the various mapping procedure (Voronoi cells, eigenvalues of the transition matrix, classical multidimensional histograms) should be performed. Further analysis on coupling the cardiovascular data with a body and ambient temperature could reveal more adverse effects, an issue that could be extremely important regarding the climatic changes. 

## Figures and Tables

**Table 1:** Statistical parameters of the source data (mean ± standard deviation).

Ambient Temperature (°C)	Drug	SBP	(mmHg)	PI	(ms)	*t*_B_	(°C)
NT 22 ± 2	Control	112.81	±19.54	179.22	±33.22	38.07	±0.29
	V1a, 100 mg	115.62	±12.17	173.74	±20.69	38.42	±0.10
	V1a, 500 mg	110.28	±15.35	184.77	±28.39	38.05	±0.10
	V2, 100 mg	119.98	±16.53	184.79	±38.30	38.54	±0.38
	V2, 500 mg	108.61	±14.79	176.16	±4.04	38.33	±0.41
HT 34 ± 2	Control	107.26	±4.19	188.63	±8.95	38.27	±0.34
	V1a, 100 mg	107.90	±10.52	197.08	±21.63	38.52	±0.26
	V1a, 500 mg	110.40	±10.07	177.21	±16.34	38.57	±0.57
	V2, 100 mg	113.26	±15.41	193.14	±30.65	38.01	±0.37
	V2, 500 mg	114.28	±6.14	184.23	±12.97	38.33	±0.47
LT 12 ± 2	Control	115.22	±5.23	164.54	±24.31	37.51	±0.43

Note: Results are presented as mean ± standard deviation; SBP: systolic blood pressure; PI: pulse interval; *t*_B_: body temperature; NT: neutral temperature; HT: high temperature; LT: low temperature.

## Supplementary Materials

The following are available online at https://www.mdpi.com/1099-4300/21/11/1103/s1, Figure S1: Effects of V1a antagonists at different time offsets between SBP and PI. Upper panels: two-dimensional signals (*SBP* and *PI* interaction, *D* = 2); lower panels: three-dimensional signals (*SBP*, *PI* and *t*B interaction, *D* = 3). Left panels: Neutral temperature. Right panels: High temperature. Figure S2 Effects of V2 antagonists at different time offsets between SBP and PI. Upper panels: two-dimensional signals (*SBP* and *PI* interaction, *D* = 2); lower panels: three-dimensional signals (*SBP*, *PI* and *t*B interaction, *D* = 3). Left panels: Neutral temperature. Right panels: High temperature.

## Appendix A. Entropy Concepts in Brief

Approximate entropy, *ApEn* [1], and sample entropy, *SampEn* [3], are tools that determine the regularity of a time series $x_{1k}\in X_{1k},k=1,\ldots ,N$, based on the existence of similar patterns of increasing length [7]. Their cross (*X*) variants measure a level of asynchrony of two time series, $x_{1k}\in X_{1k},k=1,\ldots ,\;N\;\;\mathrm{and}\;x_{2j}\in X_{2j},j=1,\ldots ,\;N$ [7], with a notable difference that *XSampEn* is a symmetric measure, while *XApEn* is not [7]. Before analysis, the time series should be normalized and centered, a procedure known as z-normalization or standard scaling. Signal stationarity is an important prerequisite; otherwise, a threshold concept would be useless and the results would be unreliable [7,18,54].

The general X-entropy estimation starts with partitioning the time series into the overlapping vectors of length *m*:

(A1) $$\begin{array}{l} •\;\mathrm{Template}\;X_{1k}^{\left(m\right)}=\left[x_{1k},x_{1,k+1,}\ldots ,\;x_{1,k+m-1}\right],\;k=1,2,\ldots ,N-m+z;\\ •\;\mathrm{Follower}\;X_{2j}^{\left(m\right)}=\left[x_{2j},x_{2,j+1,}\ldots ,\;x_{2,j+m-1}\right],\;j=1,2,\ldots ,N-m+z;\\ •\;z=\{\begin{array}{c} 1\;\mathrm{for}\;\left(X\right)ApEn\\ 0\;\mathrm{for}\;\left(X\right)SampEn \end{array}. \end{array}$$

Vector distance is defined as the maximal absolute difference of the signal samples:

(A2) $$d1\left(X_{1k}^{\left(m\right)},\;X_{2j}^{\left(m\right)}\right)=\;\underset{i=0,\ldots ,m-1}{\max}\left|x_{1,k+i}-x_{2,j+i}\right|,\;\;k,j=1,2,\ldots ,N-m+z.$$

The vectors are similar if the distance is less than or equal to the predefined threshold *r*. A probability that the time series $X_{2}\;$ is similar to the given template $X_{1k}^{\left(m\right)}$ is estimated as:

(A3) $${\hat{p}}_{k}^{\left(m\right)}\left(r\right)=\mathrm{Pr}\left\{d1\left(X_{1}^{\left(m\right)},\;X_{2}^{\left(m\right)}\right)\leq r|X_{1}^{\left(m\right)}=X_{1k}^{\left(m\right)},\;X_{2}^{\left(m\right)}\;\in \;X_{2},\;\;r>0\right\}=\;=\frac{1}{N-m+z}\cdot \overset{N-m+z}{\underset{k=1}{\sum }}I\left\{d1\left(X_{1}^{\left(m\right)},\;X_{2}^{\left(m\right)}\right)\leq r\;\right\}.$$

In Equation (A3) “^” denotes an estimate, while I{·} is an indicator function that enables a compact symbolic presentation of the counting process. It is equal to 1 if $\;d1\left(X_{1}^{\left(m\right)},\;X_{2}^{\left(m\right)}\right)\leq r$; otherwise, it is equal to zero. The value (1-*z*) eliminates the self-matching for *SampEn*.

To calculate *ApEn* and *SampEn*, it is sufficient to replace $x_{2j\;},X_{2j},\;\;X_{2}^{\left(m\right)},\;\mathrm{and}\;\;X_{2j}^{\left(m\right)}$ in Equations (A1), (A2), and (A3) with $x_{1j\;},X_{1j},\;\;X_{1}^{\left(m\right)}\;\mathrm{and}\;\;X_{1j}^{\left(m\right)}.$ For *SampEn*, it is also necessary to subtract the self-matching in (A3). A negative logarithm of the estimated probability ${\hat{p}}_{k}^{\left(m\right)}\left(r\right)\;$ corresponds [61] to the information content stored in the similarity of the template $X_{1k}^{\left(m\right)}$ to the complete time series $X_{2}$. Arithmetic averaging over all the templates yields, for *(X)ApEn*:

(A4) $${\hat{\Phi }}_{}^{\left(m\right)}\left(r,N\right)=\frac{1}{N-m+1}\cdot \overset{N-m+1}{\underset{k=1}{\sum }}\log\left({\hat{p}}_{k}^{\left(m\right)}\left(r\right)\right).$$

Ithe *(X)SampEn* approach, the summation and logarithm change the order:

(A5) $${\hat{\Psi }}_{}^{\left(m\right)}\left(r,N\right)=\log\left(\overset{N-m}{\underset{k=1}{\sum }}{\hat{p}}_{k}^{\left(m\right)}\left(r\right)\right).$$

The complete procedure is repeated for the vector length equal to *m* + 1, and with the quantity *z* in (A1), (A2), and (A3) fixed to zero. Then, *(X)ApEn* and *(X)SampEn* are calculated as:

(A6) $$XApEn\left(m,r,N\right)=\;{\hat{\Phi }}_{}^{\left(m\right)}\left(r,N\right)-{\hat{\Phi }}_{}^{\left(m+1\right)}\left(r,N\right);\;XSampEn\left(n,r,N\right)=\;{\hat{\Psi }}_{}^{\left(m\right)}\left(r,N\right)-{\hat{\Psi }}_{}^{\left(m+1\right)}\left(r,N\right).$$

Multiscale entropy (*MSE*) [8,9] and its composite improvement *CMSE* [10,11,12] are based on *SampEn* estimation repeated over the time series with increasingly coarser time resolution. The coarse graining (or downsampling) of the time series $X=\{x_{k}\}$ is performed as follows:

(A7) $$x_{CMS,l,j}^{\left(\;\right)}=\left(\overset{j\cdot \tau +l-1}{\underset{k=\left(j-1\right)\cdot \tau +l}{\sum }}x_{k}\right)/\tau ,\;\;j=1,\ldots ,fix(N/\tau ),\;l=1,\ldots ,\tau ,\;\;\;\;\;\;x_{MS,j}^{\left(\tau \right)}=\;\overset{j\cdot \tau }{\underset{k=\left(j-1\right)\cdot \tau +1}{\sum }}x_{k}=x_{CMS,1,j}^{\left(\tau \right)}$$

*CMSE* and *MSE* are evaluated as follows:

(A8) $$CMSE\left(\tau ,m,r,N\right)=\left(\overset{\tau }{\underset{l=1}{\sum }}SampEn(x_{CMS,l}^{\left(\tau \right)},m,r,N\right)/\tau ,\;MSE\left(\tau ,m,r,N\right)=SampEn\;\left(x_{MS}^{\left(\tau \right)},m,r,N\right).$$
