# Supplementary material for: Voronoi Decomposition of Cardiovascular Dependency Structures in Different Ambient Conditions: An Entropy Study
Source: Entropy (Basel). 2019 Nov 11;21(11):1103. doi: 10.3390/e21111103 (PMC7514447; doi:10.3390/e21111103)
Supplement: Supplementary file 1 [file entropy-21-01103-s001.pdf]

Supplementary material

The Figures S1 and S2 show the composite multiscale entropy *CMSE* estimates taking into account the following levels of freedom:

1. Two-dimensional signals (*SBP-PI* interaction) and three-dimensional signals (SBP-PI-t_B interaction).
2. Different *SBP-PI* offsets (delays) - 0, 1, 2, 3, 4, 5
3. Different vasopressin antagonists (V1a and V2);
4. Different dosages (100 ng and 500 ng)

Figure S1 shows the effects of V1a, Figure S2 shows the effects of V2. Thin lines correspond to 100 ng, thick lines correspond to 500 ng.

At each particular offset (delay) there exist a difference between the entropy estimates if 100 ng is administered, and if 500 ng are administered. The exceptions are three-dimensional signals with V1a at neutral temperature, three-dimensional signals with V1a at high temperature, but not for all scaling values, and three-dimensional signals with V2 at neutral temperature, for less than half of the scaling values.

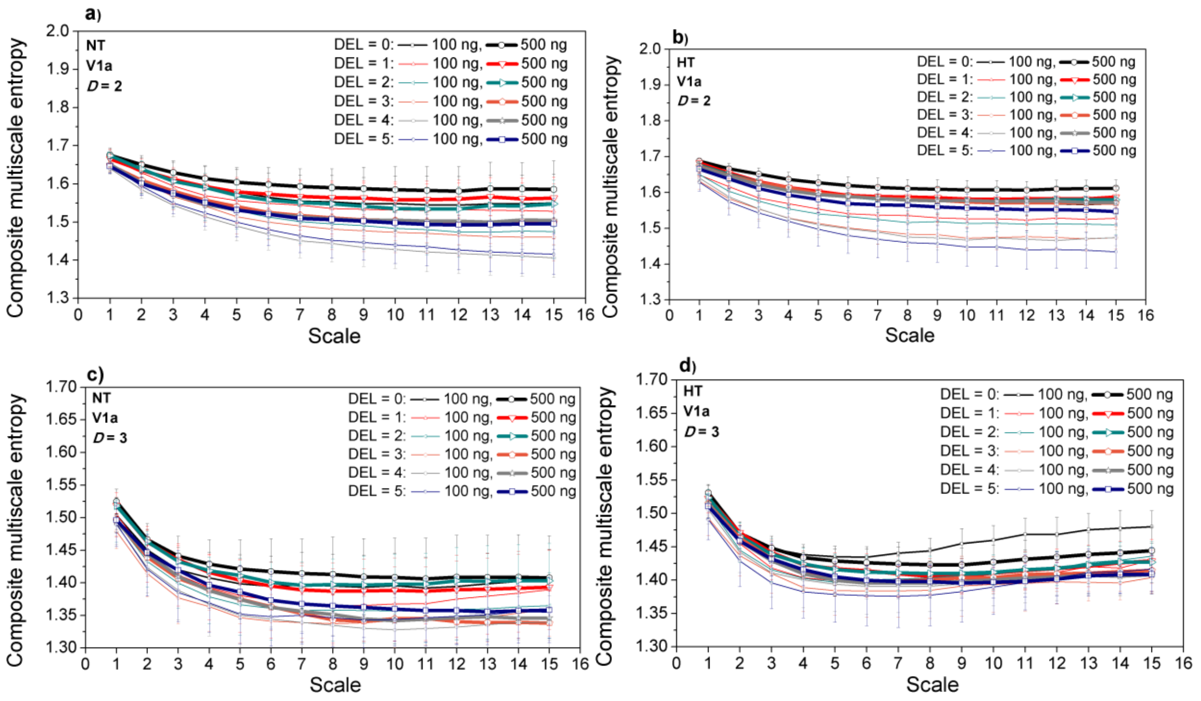

Figure S1: Effects of V1a antagonists at different time offsets between *SBP* and *PI*. Upper panels: two-dimensional signals (*SBP* and *PI* interaction, *D*=2); lower panels: three-dimensional signals (*SBP*, *PI* and t_B interaction, *D*=3). Left panels: Neutral temperature. Right panels: High temperature.

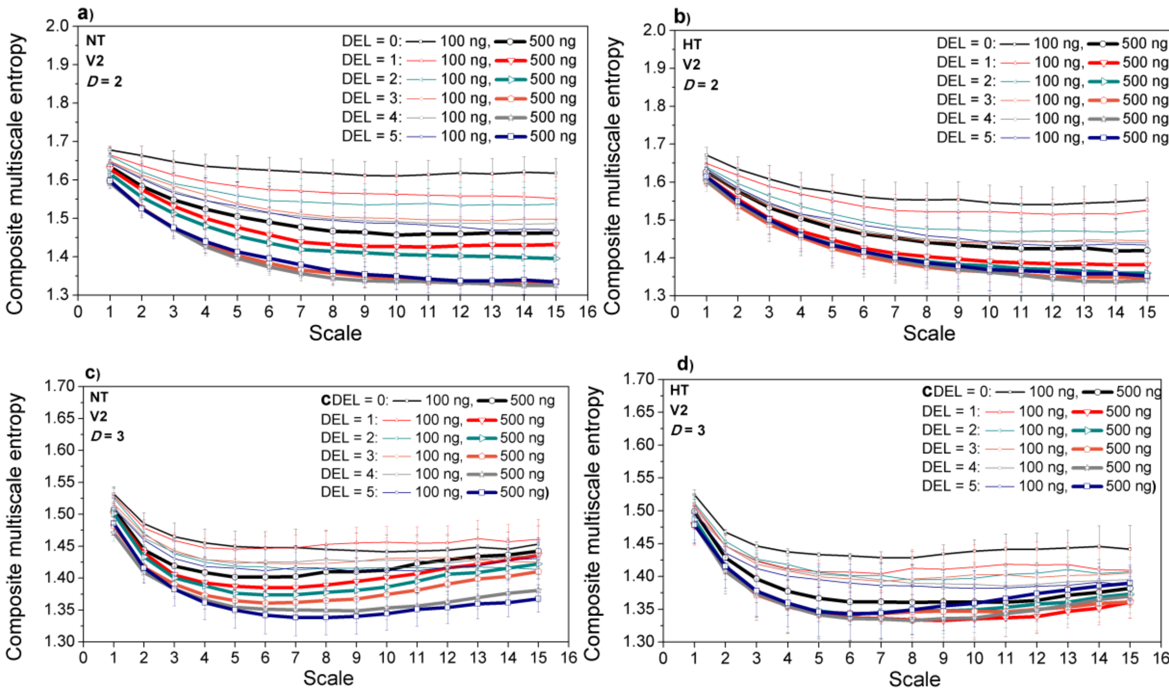

Figure S2. Effects of V2 antagonists at different time offsets between *SBP* and *PI*. Upper panels: two-dimensional signals (*SBP* and *PI* interaction, *D*=2); lower panels: three-dimensional signals (*SBP*, *PI* and t_B interaction, *D*=3). Left panels: Neutral temperature. Right panels: High temperature.
